# The Experience of Self-Transcendence in Social Activists

**DOI:** 10.3390/bs13010066

**Published:** 2023-01-11

**Authors:** Carol Barton, Rona Hart

**Affiliations:** 1 Previously School of Psychology, University of East London, Water Lane, London E15 4LZ, UK; 2 School of Psychology, University of Sussex, Falmer, Brighton BN1 9RH, UK

**Keywords:** self-transcendence, social activism, prosocial behavior

## Abstract

Every day the wellbeing of disadvantaged individuals and communities is being transformed through the activities of self-transcendent social activists. The positive contagion generated by their actions is felt globally through influence, replication, leadership training and education. These people are visionary, brave, and describe their lives as joyful, deeply fulfilled, and impactful. Seeking no personal recognition or accolade, born from a deep feeling of connectedness and a vision of how life could be better, participants describe the factors that influenced their decision to dedicate their lives to serving the greater good. Using Constructivist Grounded Theory, in-depth semi structured interviews were carried out with eight participants who self-identified as self-transcendent social activists, who have initiated non-mandated and not-for-profit community action. Data was analyzed to explore each participant’s personal experiences of self-transcendence and how being self-transcendent has manifested their life choices. The findings present a definition of ‘self-transcendent social activism’ and a theoretical model that explains the development of participants’ activism: trigger, activate, maintain and sustain, resulting in an impact experienced at three levels - individual, community and global. Theoretical and practical implications are discussed.

## 1. Introduction

The course of history has been changed by many highly impactful self-transcendent social activists who committed their lives to bring about social transformation in the communities and countries in which they lived and served. Nobel Peace Prize winner (1964), Luther-King Jr., will long be remembered for his non-violent campaign against racism that resulted in his assassination and racial discrimination being declared illegal in southern US states. Nobel Peace Prize winner (1984) and former chairperson of the Truth and Reconciliation Commission, Tutu, was influential in his campaign against apartheid and for the peace negotiations in South Africa. Whilst the legacy of Gandhi, five times peace prize nominee, whose non-violent leadership led to his assassination and to independence for India, is celebrated annually through the award of the international Gandhi Peace Prize. A review of biographical literature reveals that these courageous, visionary, people of faith prioritized freedom, equality, and the eradication of poverty above self-interest [1,2,3]. From a position of feeling connected to others and a focus that extends beyond their own personal wellbeing, self-transcendent social activists are people who act to address global problems such as inequality, poverty, environmental issues and exploitation [4].

Social activism is defined as “instances in which individuals or groups of individuals who lack full access to institutionalized channels of influence engage in collective action to remedy a perceived social problem, or to promote or counter changes to the existing social order” [5] (p. 4). Social activists are therefore individuals or groups who engage in collective action to bring attention to and resolve social problems. They operate through groups or social movement organizations that are characterized by varying degrees of formal and informal structures [5].

Self-transcendence is defined as an “increased awareness of dimensions greater than the self and expansions of personal boundaries within intrapersonal, interpersonal, transpersonal, and temporal domains” [6] (p. 179). It involves an endeavor to connect to a larger context with a prosocial intent to serve the greater good. As such, self-transcendence is a set of values and a state of mind that can prompt the motivation to engage with social activism. However, to our knowledge there is no early research that examines the connection between the two concepts qualitatively.

This study endeavors to contribute to the extant literature on the key motivations that drive social activism through the exploration of self-transcendence. Given the potential impact that activists have through the work they do in generating positive transformations in people, groups and entire societies, their goals, life choices and experiences of self-transcendence within the context of social activism is a worthy scientific undertaking.

### 1.1. Social Activism

Social activism involves taking positive intentional action and mobilizing resources to bring about change in society. Activism, both peaceful and aggressive, is expressed in many forms from writing letters, lobbying, boycotts, protests, strikes, petitions, community led initiatives, and social media campaigns. Examples of topics that social activists may engage with include environmental issues, racial equality, gender equality, refugee and immigration policies, human rights, LGBTQ+ rights, religious freedom, poverty, housing, anti-war campaigns, welfare policies, and many other topics.

Within the current Western neo-liberal social norms that emphasize individualistic pro-self goals, and independence rather interdependence, engaging in prosocial activism with a purpose of benefitting the greater good, might seem exceptional, especially since social activism is a costly endeavor, and that the chances of successful outcomes are uncertain [7]. The question of what motivates social activism, and whether it is motivated by pro-self or prosocial intents is particularly intriguing given the contrasting social norm setting.

Given the numerous social causes that social activists are engaged with, motivations will likely vary in accordance with the goal being pursued, the context, and the ideologies that underlie the activity. Recent research on the motivations of social activists has therefore aimed to elicit overarching themes to explore the underlying motives of social activists.

A repeated theme in the literature is that people might engage in activism because of injustices or deprivation that they suffered or witnessed or because they identify with the hardship of a particular group whose struggles coincide with their own experiences [8]. This suggests that critical life-events, needs, goals and interests may be key drivers of social activism [9]. An additional point raised in the literature is that negative emotions (such as pain, fear, anger or frustration) triggered by one’s sense of deprivation or injustice, or from a distressing life event, predict the willingness to engage in collective action, as well as the actual participation [10,11,12]. The perception that one’s group is negatively evaluated, disrespected, marginalized or discriminated against, can also induce willingness to engage in social action, both peaceful and violent [13,14,15,16,17].

Identity features strongly in the social activism literature as a motivating factor. National, professional, ethnic, racial, class or sexual orientation identities were found to be key drivers that motivate people to engage in social activism, and in turn, belonging to a social movement both intensifies the primacy of these identities, as well as generates a new identity that forms as a result of affiliating and identifying with the activist group [18,19,20,21,22,23].

Another motivation to engage in social action is because it renders activists personal, social or psychological benefits [24]. Gains accrued from activism include a sense of meaning and purpose, positive self-regard, belonging to a group or a community, and improved wellbeing [24,25,26,27,28,29,30,31,32]. Social activism was also associated with a feeling of personal significance [33], indicating that when people feel that they lack significance, they were more willing to pursue a political cause, at times involving violent actions, and making personal sacrifices [25,34]. Klar and Kasser [24] showed that activism was positively related to self-determination and meeting three basic needs: autonomy, competence, and relatedness. Another benefit from social activism comes in the form of positive emotions, such as exhilaration and awe, empowerment, pride, joy and sense of solidarity [11,24,35,36,37].

Values and ideologies often translate into visions and are also important motivating factors that can drive people to take social action often by eliciting a sense of social responsibility and urgency [38]. Prosocial values also play a central role in motivating action on behalf of a social cause [39,40,41,42]. Similarly, ideologies, moral convictions and religious beliefs are positively associated with activism [43,44,45,46], and acting on what one sees as core values engenders a sense of meaning in life, significance, and fulfillment which in turn elevate self-esteem [47,48]. In the context of political activism, moral convictions were found to be associated with pride [49]. Interestingly, people may adopt particular values and pursue social action due to guilt about one’s own privileges or for causing harm, or for not doing enough [50,51]. Another type of motivation that can drive social action is generativity: the desire to care about the welfare of future generations [22,52,53,54]. These are linked with other prosocial states such as empathy, perspective taking, compassion, accountability, and sympathy which have been shown as motivational factors that can prompt people into social action [55,56,57,58,59].

The brief review offered above of the factors that can motivate social activism suggests that it can be motivated by pro-self or prosocial goals, intents and attitudes, and these contrasting underlying mindsets, can impact both personal and community results. Pro-self motives can manifest in the desire to construct and maintain positive self-images of oneself as worthy and valuable, and might lead to displaying concern for others insomuch as this serves the need of the ego [60]. This can lead to the ‘white savior’ stereotype: imposing patronizing models or solutions on those in jeopardy, leading at times to the perpetuation of their condition [61,62].

In contrast, people who are motivated by prosocial motivations report empathetic identification with disadvantaged groups, experiencing acute awareness of issues that need to be changed and a belief that they can make a difference [63]. While they may sacrifice their personal time and resources, paradoxically their work may result in enhancing their own personal wellbeing [24], in addition to benefitting the greater good by attracting attention to social problems, creating solutions, and developing partnerships [64].

The link between motivation, intents and outcomes in social activism raises the question of self-transcendence as a driver of social activism. Next, we unpack the concept and briefly review the literature.

### 1.2. Self-Transcendence

Frankl [65] (p. 115) maintained that “being human always points to something or someone greater than self … the more one forgets oneself—by giving himself to a cause to serve… the more human he is…” Accordingly, within transpersonal psychology, self-transcendence involves serving a purpose greater than the self with a selfless intent [66,67]. Reed [68] (p. 397) defines self-transcendence as “the capacity to experience connectedness and expand self-boundaries in four dimensions: intra-personally by gaining more self-awareness, inter-personally by relating to others and nature, temporally by integrating past and future in a meaningful present, and trans-personally by connecting with spiritual dimensions of indiscernible world”. Other authors argued that the term signifies a developmental process whereby one’s consciousness expands beyond personal, bounded, and self-directed ego, to include other people and concerns within that sense of expanded identity [69]. Maslow’s [70] hierarchy of needs suggests that one of people’s top growth needs is the desire to reach self-actualization whereby one can realize his or her full potential. However, it has been argued [71,72] that later in his life Maslow discovered that self-actualizing individuals were capable of even higher psychological development by transcending their own self-centered goals, and pursuing higher causes that are other-orientated.

The most coherent and possibly the most cited description of self-transcendence emerged from Schwartz’s [73,74] values theory. Schwartz [73] conceptualizes values as beliefs about what is desirable, worthy and important. As such these beliefs shape one’s perceptions of oneself, others, and situations, guide one’s life goals, priorities, and decision-making, influencing related behaviors. Schwartz’s [74] values model offers a classification of two broad bi-polar dimensions, each of which incorporates several values: One axis ranges between ‘openness to change’ to ‘conservation’ values, and includes self-direction and stimulation on one side of the axis, and security, conformity and tradition on the other side. The second axis has ‘self-enhancement’ on one side and ‘self-transcendence’ on the other. It includes power and achievement on one side, and universalism, benevolence on the other side. Hedonism is placed across two dimensions: openness to change and self-enhancement. Self-transcendence values - benevolence and universalism, are characterized by a reduction in self-centeredness, and the capacity to transcend one’s own selfish needs, to care for the interest and welfare of others. As such they are considered other-focused, growth promoting values [73].

An alternative conceptualization of self-transcendence suggests that it is a personality trait linked to spirituality [75] whereby a person “identifies the self as part of the entire cosmos” [76] (p. 975), feeling a sense of connection to the universe, interdependence and responsibility.

It is also seen as a core virtue within the VIA character strengths and virtues classification, encompassing of character strengths of appreciation of beauty and excellence, gratitude, hope, spirituality and humor [77]. This trait has been associated with experiencing elevation emotions such as awe, ecstasy, amazement, worship, and flow as well as meaning in life [77].

In terms of its development or emergence, self-transcendence seems to be expressed more strongly in people who confronted difficult life experiences such as loss or illness, hence seen as a sign of adversarial growth in terms of the capacity to transcend one’s own needs and experiences, taxing as they may be, to express universalism and prosociality [78,79,80].

Further exploration of the concept suggests that it can be active or passive in terms of its behavioral manifestation. Although self-transcendence is positively associated with taking action [81], it is indeed possible for self-transcendence to remain passive.

Another finding is that self-transcendence values promote prosocial attitudes and states (such as empathy, trust, love, affection and compassion) and motivate a variety of prosocial behaviors (such as offering encouragement, care, or support) [82,83,84,85]. Some gender differences were detected, as women were found to attribute more importance to self-transcendence values while men attribute more importance to self-enhancement values [86].

There are indeed some self-benefits that people can gain from holding self-transcendence values. A positive association was found between self-transcendence and wellbeing, positive emotions, happiness, quality of life (in severely ill patients), healthy behaviors, meaning and purpose in life, self-esteem, hope, sense of coherence, mindfulness, flow, adaptive coping strategies and resilience [87,88,89,90,91,92,93,94,95,96,97].

### 1.3. Self-Transcendent Social Activism

While theoretically self-transcendence can become a strong motivator for social activism, there is little research that explores this point. In two cross-national studies [82], the authors concluded that people who hold self-transcendence values are more likely to be involved in political activism. Similarly, Gundelach and Toubøl [98] found that the values of self-transcendence were associated with activism in the context of refugee solidarity. Another study on environmental activism examined the relationship between activism and moral identity and concluded that self-transcendence positively predicts environmental activism, while self-interest values were associated with apathy leading to low environmental activism [99]. In another correlational study Hackett [100] found that the association between self-transcendence values and activist behaviors was stronger when these values emerged from personal concerns.

To our knowledge no further research has explored the association between self-transcendence and social activism, and there is no qualitative research which explores how self-transcendence is manifested in social activism.

### 1.4. The Current Study

The aim of this study was to qualitatively explore the experiences of a mixed age and mixed faith group of activists, who self-identify as self-transcendent, in order to answer the question: ‘In what way does the experience of self-transcendence manifest in the work and lives of social activists?’

In exploring this topic qualitatively, the paper aims to address a gap in the literature and contribute to our understanding of the drivers of social activism.

## 2. Materials and Methods

This study applied a Constructivist Grounded Theory (GT) approach to collect and analyze qualitative interview data [101], as a means to explore self-transcendence within the context of social activism. Grounded Theory is particularly useful for exploratory studies and its key strength is in facilitating the development of theoretical models emerging from the data. It has several distinctive features [102,103]:

● Data collection and analysis cycles: Grounded Theory involves an iterative data collection and analysis process whereby early data collection and initial analyses inform subsequent decisions on the direction and focus of data to be collected and on sampling, while the analysis remains open to new emergent topics.
● Sampling aimed at theory generation: Sampling in Grounded Theory, is initially purposive (identifying and selecting participants who are knowledgeable about or experienced with the phenomenon of interest), and later it becomes theoretically driven (known as theoretical sampling), since sampling decisions draw on early analysis and reflect the ongoing theoretical development that occurs as a result of the data collection and analysis cycles.
● Developing a theory from data: Grounded theory is designed in a way that enables researchers to develop a theory/model from data [102,103]. As such, it requires the application of inductive reasoning (bottom-up) which enables researchers to extrapolate a theory from a set of individual cases. This involves moving from the particular case to the general, and from a detailed description to an abstract level [101].
● Data analysis: Analyzing data in Grounded Theory involves applying several techniques. Initial or open coding involves analyzing the text by coding word-by-word and line-by-line and naming each segment of the data. This is often followed by focused coding which is aimed at generating conceptual codes [102]. Focused coding involves the use of some of the following techniques:
  ○ Axial coding: Relating categories to subcategories and making explicit connections between them.
  ○ Comparative coding: Constant comparisons between data in order to find similarities and differences and establish analytic distinctions.
  ○ In-vivo coding: Preserving participants’ meaning in the coding.
  ○ Selective coding: Distinguishing the core categories and connecting them to other categories.
  ○ Core categories: Identifying the components of the model/theory (they are the ones that most frequently emerge from the data, they have identifiable properties and are linked to other categories).
  ○ Theoretical coding: Specifying the relationships between categories and integrating categories to create a coherent depiction of a model or theory.

Whilst the nature of the interviewer-imposed questions meant that it was impossible to totally eliminate researcher bias, interaction between researcher and participants took the form of clean language open questions and passive listening, enabling participants to speak openly and spontaneously of their life experiences and for theories to emerge from participants’ narratives [103]. The research outcomes, therefore, are a co-construction of a theoretical model based on the data and the interpretation, observations of the first author, who, for many years, has supported Africa-based social activists through coaching and consultancy.

### 2.1. Participants

Criterion sampling (a sub-set of purposive sampling) was used in this study to define and invite the target participants. It involved searching for participants who meet a certain criteria. In this study, the key criteria was involvement in social-activism and experiencing self-transcendence. For the purpose of recruitment and self-selection of participants, the following definitions were used (see Table 1):

The participants self-identified with the above statements and satisfied the following inclusion criteria:

- Feeling connected with something greater than oneself
- They had initiated a non-mandated not-for-profit community program to reduce poverty, injustice, homelessness; the program had been operational for at least two years and positive community impact can be evidenced.

Potential social activists were identified through the first author’s personal networks, which included former colleagues, coaching and business clients. The researcher also invited former colleagues to recommend suitable participants. Prospective participants were initially contacted via an email that informed them that the researcher was seeking social activists who have experienced self-transcendence; the study aimed to explore their experience of self-transcendence and how this had manifested in their life choices.

Eight social activists, six females and two males, of mixed nationalities and religions, aged between 35 and 60 committed to participate in the study. Table 2 details their background and domain of social activism (pseudonym are used to protect their identities). No incentives were offered to encourage participation.

### 2.2. Data Collection

Following receipt of ethical approval from the first author’s University, potential participants were contacted via email. Prior to the interviews, participants were provided with more detailed information about the purpose of the research including information about confidentiality and their right to withdraw. Written consent was obtained. A draft set of questions was provided prior to the interviews.

Semi structured Grounded Theory interviews that lasted between 45 and 80 min were conducted online by the first author and recorded using Zoom. After reminding participants about the purpose of the research, interviews commenced by asking “what does self-transcendence mean to you?” The researcher used clean language [104], open questions to develop a conversation about their personal experience of becoming self-transcendent and the role that self-transcendence plays in decision making. Listening attentively for themes and insights, the researcher asked more probing follow up questions to stimulate deeper reflection about specific characteristics of self-transcendence and what factors strengthen or weaken their experience of self-transcendence. Example questions include: What does the term self-transcendence mean to you? Thinking about your own experience of becoming self-transcendent, how would you describe that? How has being self-transcendent influenced your life choices? How does being self-transcendent manifest itself in your social activism? In other areas of life? What are the benefits and challenges of being self-transcendent?

Whilst one participant described in some detail her personal experience of becoming self-transcendent, other interviewees steered the interview in the direction of how being self-transcendent has motivated and influenced their life choices, and how this manifests in their pursuit of social activism. The resulting theory, therefore, represents a ‘self-transcendent’ infused model of social activism.

The study followed Grounded Theory guidelines by conducting cycles of data collection followed by initial analysis which entailed line by line coding [102]. This meant that between interviews, data were coded, and key themes identified for deeper exploration were introduced through focused questions in subsequent interviews. For example, in early interviews ‘courage’ and ‘empathy’, emerged as important themes leading to more exploratory questions in later interviews.

Whilst time constraints meant that the number of participants and interviews was limited, the sample size was considered large enough for a robust theory to emerge [105], and data saturation was achieved within this sample size and interview framework.

### 2.3. Data Analysis

Interviews were transcribed using a transcription service and manually checked to ensure verbatim accuracy. This enabled the researcher to gain an in depth understanding of the data. As noted, data collection and initial analysis (open coding) occurred simultaneously. Once open coding was complete for all transcripts, several types of focused coding techniques were applied to create a more abstract analytical framework [102]. The first stage included sorting the numerous themes that emerged from the initial coding, to identify and focus on the most salient ones [102]. Then axial coding was applied as a means of linking between categories and their subcategories, some of which readily emerged from the text. Comparative coding followed and involved comparing categories across different segments of the data in order to find similarities and differences and to establish clearer distinctions between elements that initially seemed to be entangled together [103]. The next stage involved selective coding. At this stage it became clear that the focus of the model would be around the process of becoming self-transcendent social activists. This stage held the key to reducing the number of categories and focusing the analysis on the most significant ones which were eventually identified as the core categories [102,103]. The last stage involved theoretical coding - refining the categories, specifying the relationships between them, and integrating them into a coherent model [103].

In order to produce a visual representation of the emergent model, the data were then imported to NVIVO for further analysis. Earlier work by Bazeley [106] and others [107,108] demonstrated the usefulness of NVIVO in facilitating a grounded theory analysis. Hutchison, Johnston and Breckon [107] argued that the benefit of NVIVO is in providing a transparent account of the analysis process which enhances its rigor. Although NVIVO can be used to conduct all stages of the Grounded Theory analysis, in this study it was only used to help generate a clearer account of the model.

The conceptualization of a theoretical model of ‘self-transcendent infused social activism’, enabled the researcher to refine, condense, and align the data to the final six themes which are described below.

## 3. Results

What started off as an investigation into the experience of self-transcendence in the lives of social activists became a broader discourse about what motivated participants to commit their lives to activism, the impact this has had on their personal lives and the communities they serve and more globally. The analysis of data resulted in the emergence of:

1. A definition of self-transcendence within this context
2. A description of how self-transcendence activism has impacted the lives of participants and the people they serve
3. A model comprising four continuous stages of activism - trigger, activate, maintain and sustain.

These are summarized in Table 3.

### 3.1. Definition

The definition domain describes how the participants responded to the question ‘what does self-transcendence mean to you?’.

#### 3.1.1. Feeling Connected to Something Greater Than Oneself

Without exception, Christian, Hindu and Muslim participants expressed the importance their faith, combined with a commitment to live a life aligned to their spiritual convictions:

*‘It’s definitely, my Christian, commitment and wanting to walk and do things for others’ (Jemma).*

*‘I’m very strong in my faith, but …I don’t want to force that on other people. But I also make sure that I live my life in the values of my faith and that helps me in terms of how I walk and interact with the community’ (Sam).*

*‘When I walk in my calling, directed by God (Fiona)’.*

Connection to something greater also included the concept of seeing oneself as part of a bigger community, connected to all humanity:

*‘A small cog in a large wheel’ (Sam),*

*‘As individuals we are not complete in our separateness’ (Natalie).*

*‘There’s another expression that says ‘you are because we are’ so you always understand that your life is connected to others….’. (Fiona)*

#### 3.1.2. Self-Awareness

Most respondents noted that self-awareness and self-care are precursors to self-transcendence and the process of becoming self-transcendent involves self-reflection, self-knowledge and healing. To help other people in a healthy, safe and benevolent way, first it is necessary to become a ‘safe person’:

*‘…in my process of transcendence, part of my journey was understanding who I am. I think you cannot transcend yourself if you haven’t taken care of yourself. So, there’s an element of understanding yourself, growing and knowing who you are’ (Fiona),*

*‘And then there’s your own growth as a human and your own sort of evolving identity that sort of interacts with that... it is a process because you have to continually answer the question of what is actually happening around me, how do I interpret it? How do I make meaning out of the things I’m seeing?’ (Tandy)*

#### 3.1.3. Increased Awareness of Social Justice Issues

The majority of the participants reported a heightened awareness of inequality, poverty and other prejudices combined with a belief that the situation can be improved. Whereas other people might not be aware of injustices, participants reported both noticing and wanting to respond to inequitable access to resources and opportunities:

*‘It makes you aware of other people’s lives, other people’s struggles. God put compassion and empathy in you, and you can’t limit that compassion and empathy to just a small group of people’ (Judith).*

*‘It’s how we view the world, how we value things. I cannot sit back and see somebody else being in total despair’ (Fiona).*

#### 3.1.4. Reduction in Self-Interest

Whilst we all need validation, if affirmation, personal gain or enhanced self-esteem is the motivation; that is not self-transcendence, and this was noted by several participants. The participants also noted that in self-transcendence, the focus and concern are no longer on self but on the people being served. When self-gratification desires reduce there is a much greater sense of freedom:

*‘You’re doing things not just for your ego, not to be noticed. You don’t need public acclamation. and you’re not doing it for personal gain. Doing it out of love and compasion—There is something deeper within you’ (Jemma).*

*‘So basically, it’s about putting others first rather than putting yourself first.’ (Tim)*

#### 3.1.5. Desire to Be of Service

The act of serving others was mentioned by several participants who considered it much more satisfying and rewarding than doing things just for oneself. To serve others brings great personal blessings, to see the smile on the face of someone you’ve helped, or just to experience the privilege of serving others, counts for so much more than self-gratification:

*‘There can be so much emptiness in just trying to self-gratify. There’s only so much you can do to self-gratify, but so much joy when you serve others and you see others happy’. (Jemma)*

### 3.2. Impact

#### 3.2.1. Personal Impact

The work of an activist can be demanding and grueling, but participants overwhelmingly described their lives as joyful, fulfilled, aligned to calling, abundant and meaningful. Giving joy to others is described as contagious, great fun, extremely rewarding and this creates a desire to do more:

*‘..it can just be exhausting. Honestly just to be so empty, you know….as the social activist, learning to give your life away, and when you really look at what it definitely includes, bringing fulfilment, and when you are completely exhausted, exhausted for the social good.... it gives me a lot of joy. It’s grueling but it gives me joy’ (Jemma)*

*‘Yes, it does require personal sacrifice. But for me, I don’t see it as personal sacrifice because I enjoy doing what I do and I see it as an opportunity, I derive a lot of joy. So, for me I count it as a privilege …it makes you want to do it more because you get joy in other people’s joy. I think joy is contagious, and so, giving joy is just so much fun’ (Tina).*

*‘Fulfilment, deep fulfilment, challenging, rewarding.’ (Judith)*

#### 3.2.2. Community Impact

Eight community programs are represented across four continents. Participants reported working with victims of HIV, disabled children and their families, the homeless and people living in slums to provide education, medical services, social care, adult skills and employment training, mediation, infrastructure support, leadership development, affordable housing and other initiatives to alleviate poverty and empower communities:

*‘It began growing organically because when you support a woman she comes with the entire family. A woman comes with children, youth, adolescents, and she brings the community. And as a result, she also came with sickness and this affected the education of the children and became an issue. Socioeconomic empowerment is an issue we’ve been tackling initially as well. We wanted to see how we can support her to earn. You’re putting that wholesome completeness in that home. So, we began by offering economic empowerment, then education for the children, then the technical certificate for their older children. We were training women to do different skills and assessing their credit to start little businesses. So that’s how the whole project started…….’. (Jemma)*

*‘We work with children with disabilities and their moms. There is no help in this country for families that are struggling with that. Every child that comes to our therapy center, comes with a mama and we provide each mama with employment ……Our heart is for people that are struggling with disabilities and their families. We work with a lot of HIV positive families and they’re just dealing with a lot of problems besides the disability. There’s so many other problems that come along when you live in poverty. But it’s always a thrill to be able to help somebody’. (Tina)*

#### 3.2.3. Global Impact

Most participants talked about the ripple effect which has been created through developing international leaders, training others within existing programs, permitting replication (at no cost) of their community development model, extending their work internationally. One participant spoke of being invited to speak to UN representatives about his work to support the homeless:

*“God has been good in my life, putting me into this position where I can be influential to a lot of people as an affordable housing developer. I’m a newbie in this industry, but I’ve been recognized as the best affordable housing developer in town. And even United Nations got a hold of my story and my philosophy as a developer... So instead of just working on developing buildings for people for the money, I follow the need of people. So my focus is to work with people, find out the need. And that’s one of the reasons why I flew to Nairobi and I saw even greater need compared to Hawaii, because they’re in need of a half a million apartments for the 3 million people who live in slum…And besides being a developer, I created a non-profit organization. And we’re reaching out to Cambodia, to the Philippines. And now I’m thinking about reaching out to Tanzania’. (Todd)*

### 3.3. Triggers

This category refers to the life experiences that set participants on a course of taking action:

#### 3.3.1. Early Role Models and Exposure to Social Injustice

All participants described how the influence of early role models, and the environment in which they were raised, shaped their outlook and made them more sensitive to injustices and inequalities:

*‘I grew up seeing my parents caring for other people, serving more than to be served and that’s how I grew to know life’. (Jemma)*

One respondent noted how her experience of a difficult childhood led to a sense of separation, fear and isolation which prompted a spiritual search for reconnection with something greater, triggering a desire to help others (Natalie). Another recalled his experience of being raised in an institution:

*‘It was shaped with my upbringing growing up in a children’s home which had more than 110 children. It’s not easy growing up in institutions - life was not easy. So that shaped my thinking about how I wanted to live my life’. (Sam).*

Exposure to social justice issues, such as homelessness, apartheid, refugees, triggered an early response and determination to take action:

*‘We had refugees in our home, and you are meant to take care of them. I saw my dad bring one - he was an Ethiopian refugee when there was war in … and then when I was in the university myself, I brought in a refugee, I’ve always had that desire to reach out to people who are either homeless or suffering and to serve them’. (Jemma)*

#### 3.3.2. Personal Experience of Tragedy

Experiencing personal tragedy, or seeing tragedy close up often triggered negative emotional and behavioral responses; however, for our participants experiencing trauma it triggered a motivation to help others:

*‘Our son was born at 22 weeks. He survived many heart attacks and we saw him come back to life many times after having no heartbeat. And, he was a true miracle. and that was my baby…… and then God asked me to do a special needs ministry’. (Tina).*

*‘It was the first time I saw a mother and a child laying on the side of the street and I was in complete shock. Like I couldn’t believe that traffic wasn’t stopping, and people weren’t helping her. Like it was such a foreign concept to me. and that definitely was a trigger too.’. (Judith)*

#### 3.3.3. Feeling ‘Called’ or Compelled

Six participants reported a sense of calling, feeling compelled, or hearing from God, to which, in spite of the personal sacrifices demanded and not knowing where resources might come from, triggered a conviction to respond. One participant reported seeking God’s will through prayer and reading the Bible:

*‘God has called me to serve the very disadvantage, very poor, in the slum…. So out of obedience to God he called me to go into that community and walk alongside women like that’. (Jemma)*

*‘It’s a calling from God truly that he’s asked us to do this. I know that sounds, for some people kind of weird, but it is definitely what we feel called to do. Now, did I hear a voice when I say the word calling? No, but I spend a lot of time, praying and reading the Bible and asking God to keep directing us.’. (Tina).*

*‘There is the compelling and choosing not to ignore that compelling. God spoke to me and I know for certain that I heard the call and we responded’. (Judith)*

### 3.4. Activation

These themes moved participants from ‘making a decision’ to take action by the operationalization of that decision.

#### 3.4.1. Empathy, Compassion and Connection

Common themes running through all interviews were how compassion and empathy led to taking action. Empathy enables one to identify and connect with the community, as opposed to sympathy which can be seen as adopting a superior position and imposing solutions. Feeling compassionate often draws one into becoming deeply empathetic.

*‘When you talk about transcendence, transcendence is not about sympathy. It must have empathy. If empathy is not in you then you’re totally missing the point. So, empathy enables you to identify and connect with the community. Whereas sympathy puts you on a higher position and you’ve got power. Sympathy is all about listening with your head. But empathy is about listening with your heart’. (Sam)*

*‘So, my job I believe is to inspire all these people that there is a choice that we can make to have compassion and empathy for other people who are less fortunate than them/me’. (Todd)*

#### 3.4.2. Courage and Faith

Without courage, self-transcendence can remain passive. All participants spoke of the need to exercise courage, an internal quality that you carry on the inside–courage to admit one does not know all the answers, to be unpopular, to travel across the world and live in dangerous places, and courage to take risks. The notion of faith includes believing that resources will be provided, and things will work out whilst the path remains unclear:

*‘For sure you can’t do what we do without courage. You need both self-courage and you just need overall courage. ….I want to learn the courage to say I’m not here to help. I’m here to walk with you and everything. and even the courage to have a brave face to go into hard places.’ (Sam).*

*So you have to sacrifice something in order to be courageous and to step up and do, especially when you’re trying to help other people. You gotta have courage’. (Todd)*

#### 3.4.3. Having a Vision

Participants reported observing patterns and seeing life through a lens of possibilities. Rather than looking at problems and what does not work, starting from the position of appreciating what works, seeing potential in others—what they are capable of becoming and having a visualization of what might be:

*‘And I always say, because it is God’s work, he provides the resource, it’s his vision’. (Jemma).*

*‘I was primed to see things in a way that would make me want to do something about it. …….. for several months prior to the vision trip that I took’ (Tandy).*

*‘So you have a vision. It’s challenging, but it’s also extremely rewarding, because I’ve been doing it for some time, like for instance our rescue centre in Romania, those kids are now grown’. (Judith)*

### 3.5. Maintain

The life of an activist can be demanding and exhausting. The resolve to remain committed is strengthened through several factors:

#### 3.5.1. A Community of Like-Minded Individuals for Support

Surrounding oneself with a supportive circle of encouraging, like-minded people who act as co-mentors increases motivation and provides opportunities to work collectively:

*‘If you have healthy intimate relationships and strong connections with other people, there’s an exchange - you’re learning with other people, you’re serving with other people. I think that increases self-transcendence because you get the opportunity to watch other people being courageous’ (Fiona).*

*For family members, the support of a partner and family to cheer you on is vital:*

*‘I don’t think that God’s going to call me one way and my husband another way because we are in this together as a married couple. and so, we make decisions together’. (Tina)*

#### 3.5.2. Personal Sacrifice and Self-Care

The importance of exercising self-care, taking breaks and time out, spending time with family, spiritual connection and devotion were reported as being important to maintain good emotional, spiritual and physical health:

*‘I made sacrifices thinking that I could withstand it, thinking that my marriage could withstand it. I’ve made a lot of sacrifices. I think first of all, money, it took me five years, before I launched xxx…..And so that’s a very concrete data point around the financial cost’ (Tandy)*

*‘I want to have more time with my daughter. I kinda need to start being selfish myself. That’s called self-care and boundaries.’ (Sam).*

*‘ self-care is obviously very important. Having healthy boundaries is really important…. So, I have to go to the source, which is God, he has an abundance. So, if I’m not going to the source, it’s like not plugging my computer battery in. It’s not going to last very long’. (Judith)*

*‘And of course, in this kind of work, you really have to know how to take care of yourself. I’m here trying to recover. Cause the last week I was working so much, but I am happy.’ (Jemma)*

#### 3.5.3. Seeing Possibilities and Co-Production

Co-production is when a community comes together to influence and design policies and services that benefit all, rather than becoming consumers of solutions supplied by non-community members. This approach creates a sense of interdependence and connectedness whereby people develop confidence to care for each other and co-create solutions. Co-production is seen as an essential factor in maintaining programs and accomplishing community empowerment. For many participants, this has involved exchanging western comforts to live in a Nairobi slum, to truly understand what this feels like on a day-to-day basis:

*‘Once you start putting that community in a box and you’re not within that box you’re outside, then you’re not in the community, then that’s a problem. You’re not actually working with the community - you are working against the community. Or, you’re actually looking in terms of “how do I bring a fix” with me?’ (Sam)*

*‘I think that just being at the same level with everybody here is an important piece. Living with them, working side by side, shoulder to shoulder, trying to understand what they’re going through, even though ultimately I can never fully understand’ (Tina)*

### 3.6. Sustain

Participants expressed a desire to see the life of a self-transcendent activist become more commonplace, describing the possibility in terms of ‘heaven on earth’ or utopia, a world filled with more justice, equitable opportunities and resources, joy, compassion, gratitude and kindness. Poverty, oppression and greed would be reduced. Important factors that lead to sustaining impact and growth are identified below:

#### 3.6.1. Having a Global Perspective

Technology and media support a sense of connection with people all over the globe. Problems experienced by individuals, communities and countries are no longer viewed in isolation and participants reported how recognizing the interconnection of all things leads to the development of global solutions and co-operation that grows organically, often from something small to something that has global impact:

*‘We seem to have embodied this ethos on a global scale because we have kids from all over the world’ (Fiona)*

*‘What I do - I offer up and create and hold space for entrepreneurs to also discover their own purpose and their own capacities and their own power’ (Sam)*

*‘Because the more people that are connected and understand this and are able to move outside of themselves, the better society is because then everybody, everybody becomes a resource but in a positive way, not in an exploitative way, but in a synergistic way, like in a way that that brings beauty to society’ (Fiona)*

#### 3.6.2. Growing Leaders

Leaving a legacy means training the next generation of leaders. Where this is neglected, the potential impact of initiatives is not sustainable. An example offered by one participant was of a situation where an influential community leader who had initiated many community programs, unexpectedly died before training successors. His death resulted in a fight for leadership and political chaos:

*‘He was able to develop so many other things, but he failed in one thing. He failed in grooming leaders to take over from where he was. So indirectly you can say he was self-centered in his leadership because if he had intentionally groomed other leaders, we would not be having the chaos we are having with the political parties’ (Sam).*

*‘What I’ve done mostly I’ve chosen to mentor others then meet other people who are committed and have the same heart and the same calling. Increasingly, I’m investing my time doing that mentoring, coaching so that more people can develop that attitude.’. (Jemma)*

#### 3.6.3. Teaching Empathy, Awareness and Courage

Without courage, self-transcendence can remain passive. According to Sam, ‘without empathy, you’re missing the point’. Self-reflection, self-knowledge and healing are necessary precursors to helping others. Awareness of social justice issues is a trigger for many activists. Embedding the concepts of empathy, self-awareness, awareness of social injustice and courage into the educational, mentoring and coaching methods deployed by participants and their organizations was reported to be a high priority:

*‘There are others that are coming behind me that I need to teach and I need to teach them to be courageous’. (Fiona)*

*‘There needs to be a way in terms of how we start breaking those walls and start having conversations in terms of me and you, this is where I come from and where you come from. Not based on tribe ethnicity or your race or your religion - then we start developing empathy in a different way. So my priority now is I’m doing more in terms of one-to-one where people just want to have a conversation’. (Sam)*

The resultant model brings these themes together in a continuous process of self-transcendent infused social activation which results in individual, community and (in the case of participants) global impact.

## 4. Discussion

In the midst of alarming news about escalating and urgent global problems, where every day millions live without adequate food, water and sanitation; children die from malnutrition, HIV kills thousands of people, increased carbon dioxide and other human-made emissions injure the planet and human activities create a wave of extinction of plants and animals, the lives of individuals and the well-being of disadvantaged communities is being transformed through the activities of impactful self-transcendent social activists. The positive contagion of their actions is felt globally through influence, replication, leadership training and education.

Experiencing notable levels of eudemonic wellbeing [109] participants describe their lives as joyful, deeply fulfilled, privileged, spiritual and meaningful. Leading meaningful lives sensed as a calling, seeking no personal recognition or accolades, born from a deep feeling of connectedness and a vision of how life could be better, participants described what motivated them to ‘focus on what really matters’ (Jemma) by committing their lives to a self-transcendent purpose directed towards serving others [65].

**Figure 1:**
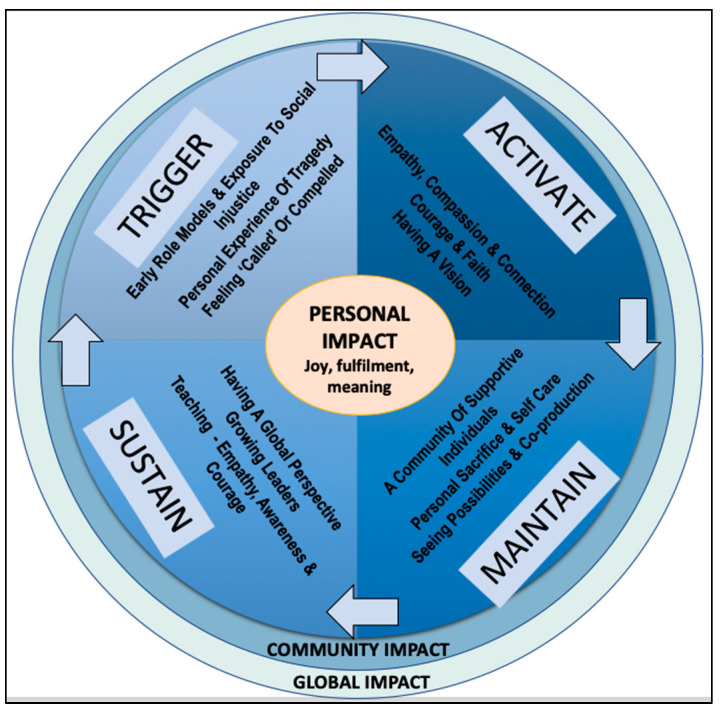
Self-transcendent social activism.

What started off as an exploration into the experience of self-transcendence within the context of social activism, led to the emergence of a ‘self-transcendence infused’ values driven model (see Figure 1) of social activism that describes four key processes—trigger, activate, maintain and sustain. The model presents a continuous process of activism that generates personal joy fulfilment and meaning whilst creating a ripple effect of positive contagion that can be leveraged to address community and global issues.

### 4.1. Self-Transcendent Social Activism

A combination of early role models, exposure to social injustice, personal experience of tragedy and feeling ‘called’ triggered a resolve to help others; findings that are align to research carried out by Dutt and Grabe [110]. Empathy, compassion, a sense of connection, courage and faith moved participants from simply having a vison of how life could be better, to take action. Maintaining social activism requires sacrifice and is challenging; participants listed a number of factors that enhanced their commitment and motivation including being surrounded by a community of like-minded individuals for support [111], willingness to make personal sacrifices, self-care, seeing possibilities rather than problems and adopting an empathetic approach that empowers communities. Sustaining momentum, so that the ripple effect of their activism reaches new communities and future generations and becomes more universally contagious, involves having a global perspective, growing leaders, and embedding the concepts of empathy, awareness and courage into coaching, mentoring and educational organisation systems.

### 4.2. Context

**Figure 2:**
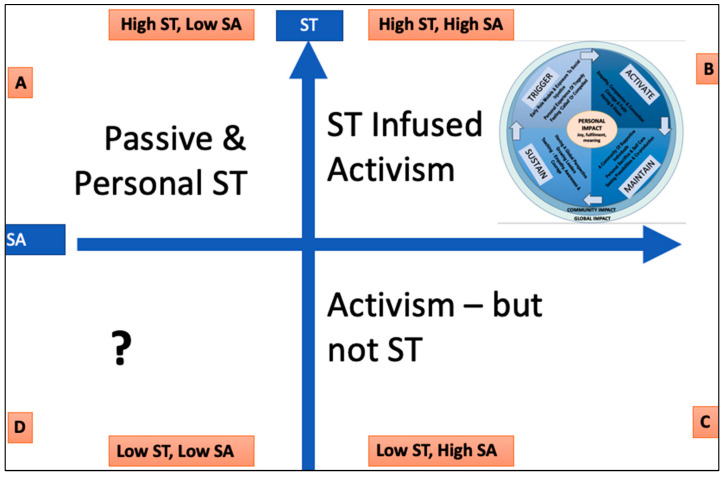
Self-transcendence + social-activism = impact.

The study results have broader implications as shown in matrix below (Figure 2), which depicts comparative levels of activism and self-transcendence.

Initiated in Hawaii, the approach taken to develop housing projects for the homeless has been extended to Cambodia and Kenya and is recognized by the UN. The approach that led to the creation and organic growth of a center of educational, medical and social support facilities located in a Kenya slum emaciated by HIV, is being multiplied through a ‘franchise’ type methodology and mentoring like-minded activists. A program which started many years ago in Romania, to rescue orphans, has led to a similar program being brought to Kenya. These are examples of how the influence of participant’s activism extends well beyond local communities. Participants self-identified as self-transcendent social activists thereby occupying quadrant B on the matrix above. High self-transcendence combined with high social activism has led to the development of sustainable co-produced community enterprises [60,64]. Here, a number of factors have coalesced, resulting in significant community and global impact. By fully identifying with disadvantaged communities, working alongside them, contributing much needed resources and skills, empowering, training and co-producing sustainable initiatives, participants have delivered significant results.

Quadrant A represents non-activated self-transcendence where the impact of a self-transcendent lifestyle remains individualistic. Feelings of connection to something greater than oneself and the motivation to do something meaningful are incubated before being activated. Life for research participants commenced in this space as self-awareness, awareness of injustice, and a desire to be of service increased. Feeling empathetic, compassionate and connected, believing they had a role to play in helping to improve the lives of others, exercising faith and bravery, overcoming challenges to pursue a goal or conviction [112]; participants moved from quadrant A to quadrant B by demonstrating commitment and a willingness to step out of comfort zones and confront challenge [113].

Quadrant C represents a form of activism that is not infused with self-transcendence values. Often more ego than eco driven, and sometimes driven by entrepreneurism and a desire to generate profit, frequently less impactful ‘solutions’ are imposed rather than co-created and are short-lived [60,114].

The research did not involve collecting Quadrant D data, which represents low self-transcendence and low activism; however, from spiritual literature [115], we may speculate that, for some, this is a lonely position, possibly with high levels of neuroticism and alienation [116] representing potential ground for further social activism.

Self-transcendent social activism, which involves the integration of ego and eco goals is highly impactful. This form of activism leads to the development of co-produced sustainable initiatives and solutions that empower local communities and create positive contagion. In comparison, non-self-transcendent activism, often motivated by personal agendas, and the need for personal recognition leads to ‘outsider’ imposed, less sustainable, models and often causes resentment. Self-transcendent activism operates from a position of ‘empathy’. According to Sam, ‘empathy involves listening with the heart, whereas sympathy involves listening to the head.’ ‘If empathy is not in you then you’re totally missing the point’.

Passive self-transcendence may benefit an individual; however, increasing societal impact involves transitioning from passive to active self-transcendence. Amongst other things, moving from passive to active requires developing a vision of how life can be better [63], believing one can make a difference, and having courage. Courage can be taught [112]. The implications and application of this study are far reaching. The study suggests that teaching and modelling empathy, compassion and courage and embedding each stage of the ‘self-transcendent social activism model’, into coaching, mentoring and educational interventions will result in increased positive individual and community impact, generating a ripple effect of positive contagion which can be leveraged to address global challenges.

### 4.3. Limitations and Future Research

A number of limitations of the current study should be considered when examining the results and conclusions. Findings were based on eight interviews with participants who self-identified as self-transcendent social activists. A limitation of the study was the predominance of female (6), and Christian (6), participants.

Within the scope of the interviews, arguably, data saturation was achieved, and no new information emerged from latter interviews. However, given more time, it would be possible to increase the sample size and to extend the range of interview questions. The researcher has attempted to eliminate personal bias; however, a number of participants were known to her. Future research, deploying a quantitative methodology to evidence impact and the use of scales to measure the relationship between transcendence, activism and wellbeing would strengthen findings.

Researching activism within the context of quadrant C—to include volunteerism, entrepreneurialism, and career activism would prove insightful.

Furthermore, testing the model in terms of training, taking before and after measurements to evidence the effectiveness of interventions designed to develop empathy, compassion and courage, is suggested by the researcher.

## 5. Conclusions

This study contributes to the extant of the literature by expanding our understanding of self-transcendence as a driver of social activism. It has resulted in the development of a new model of ‘self-transcendent social activism’ containing four key processes: trigger, activate, maintain and sustain engagement with social activism.

## Figures and Tables

**Table 1:** Definitions used for purpose of recruitment.

**Self-Transcendence**
A shift in focus from self (ego) to others; A shift in values and willingness to sacrifice self-interest to serve the greater good; An increase in moral concern and courage to act and take risks, aligned to moral compass
**Social Activism**
Non-mandated and not-for-profit practical action carried out by individuals or groups, to solve societal problems and bring about change for the good of others.

**Table 2:** Participant demographics.

Pseudonym	Gender	Nationality	Country of Residence	Religion	Context/Projects
Fiona	F	Swazi	Kenya	Christian	Pastor/Spiritual healer, laying the foundation for an international network of home educators
Jemma	F	Kenyan	Kenya	Christian	Bringing hope to poor communities affected by HIV/AIDs by providing education, medical and social care
Tina	F	American	Kenya	Christian	Providing education, medical and social support services for children with disabilities and employment training and opportunities for their mothers.
Sam	M	Kenyan	Kenya	Muslim	Eisenhower Fellow, developing local leaders, catalysing positive change, and alleviating poverty in the largest Kenyan slum
Todd	M	Filipino	Hawaii	Christian	Youth Pastor, building affordable housing units to support the homeless in Hawaii, Cambodia and Africa
Natalie	F	British (Tobago origin)	UK	Hindu	Teaching Meditation, peace circles and wellness programmes in US, India, UK and Virgin Islands
Tandy	F	Chinese American	USA/Kenya	Christian	Empowering teachers and transforming schools *in* Kenya through leadership training, instructional coaching and infrastructure support.
Judith	F	American	Kenya	Christian	Rescuing and equipping orphans and destitute children in Kenya and Romania

**Table 3:** Summary of Results.

**Definition**	Feeling connected to something greater than oneself
	Self-awareness
	Increased awareness of social justice issues
	Reduction in self-interest
	Desire to be of service
**Impact**	Personal impact
	Community impact
	Global impact
**Triggers**	Early role models and exposure to social injustice
	Personal experience of tragedy
	Feeling ‘called’ or compelled
**Activation**	Empathy, Compassion and Connection
	Courage and faith
	Having a vision
**Maintain**	A community of like-minded individuals for support
	Personal sacrifice and self-care
	Seeing possibilities and co-production
**Sustain**	Having a global perspective
	Growing leaders
	Teaching empathy, awareness and courage

## Data Availability

Data supporting the reported results is kept by the first author.
